# *Microtus arvalis* and *Arvicola scherman*: Key Players in the *Echinococcus multilocularis* Life Cycle

**DOI:** 10.3389/fvets.2017.00216

**Published:** 2017-12-13

**Authors:** Olivia Beerli, Diogo Guerra, Laima Baltrunaite, Peter Deplazes, Daniel Hegglin

**Affiliations:** ^1^Institute of Parasitology, University of Zurich, Zurich, Switzerland; ^2^Laboratory of Mammalian Ecology, Nature Research Centre, Vilnius, Lithuania

**Keywords:** *Arvicola scherman*, *Echinococcus multilocularis*, intermediate hosts, life cycle, *Microtus arvalis*, parasite reproduction, predation, *Vulpes vulpes*

## Abstract

A broad range of rodent species are described as potential intermediate hosts for *Echinococcus multilocularis*, a wide-spread zoonotic cestode causing alveolar echinococcosis. However, little is known about the relative contribution of these species for parasite reproduction and the maintenance of its life cycle. In a comparative study in a high endemic region in Zurich, Switzerland, we investigated prevalence rates and fertility of *E. multilocularis* in the most abundant vole species as well as the predation rate of foxes on these species. To ensure that the fox families had access to different vole species and that these voles were exposed to the same environmental contamination with parasite eggs, we selected eight study plots where at least two rodent species co-occurred. The parasite prevalence in *Microtus arvalis* [11.0%, confidence intervals (CI) 8.9–13.4] was significantly higher than in *Arvicola scherman* (5.3%, 3.9–7.1) and *Myodes glareolus* (3.9%, 2.0–6.7). None of the, only 29 individuals of, *Microtus agrestis* was infected (0%, 0.0–9.8) and the species was excluded for further analyses. Logistic regression models for the prevalences revealed significant differences between nearby study plots and higher infection rates for females, heavier individuals, and individuals trapped during spring, when the prevalence in *M. arvalis* peaked up to 65% (CI 50–79) in one plot. Furthermore, we detected significantly higher percentages of fertile infections in *M. arvalis* and *M. glareolus* than in *A. scherman* (OR 11.2 and 6.4, respectively) and a significantly higher protoscolex number in *M. glareolus* (median 100,000) than in *M. arvalis* (13,500) and *A. scherman* (4,290). The most abundant fox prey remains were of the genera *Microtus* (12.3%, CI 8.4–17.2) and *Arvicola* (11.5%, 7.7–16.3), whereas *Myodes* was never recorded as prey (0.0–1.3%). We conclude that *M. arvalis* and to a lesser extent *A. scherman* can be regarded as key intermediate hosts in Western and Central European high-endemic regions whereas *M. glareolus* and *M. agrestis* play a marginal role. We, therefore, postulate that distribution models of these species could contribute to predict parasite occurrence on a more detailed spatial scale than models of the distribution of foxes which have a very broad and uniform distribution.

## Introduction

*Echinococcus multilocularis* is a wide-spread cestode causing human alveolar echinococcosis (AE), a severe disease, with canids (mainly red foxes, *Vulpes vulpes*) acting as final host (1, 2). A wide variety of small mammals are described as intermediate hosts (1, 3). Individuals of some murid species were occasionally detected with *E. multilocularis* infections [e.g., one *Mus musculus* (4), one *Rattus norvegicus* (5)], but their role as intermediate hosts can be neglected (1). Regular records of rodent populations with relevant prevalences are only reported from cricetid species, e.g., *Arvicola scherman* (formerly *A. terrestris*) and *Microtus arvalis* in Europe (6, 7); *Myodes rufocanus* in Japan (8, 9); *Ellobius tancrei* and *Lasiopodomys brandtii* (Brandt’s vole, formerly *Microtus brandtii*) in Central Asia (10); *Peromyscus maniculatus* and *Microtus pennsylvanicus* in north central of the United States (11, 12).

Due to successful rabies vaccination, increased supply of anthropogenic food resources, and changing human–wildlife interactions [e.g., urban tameness (13)], fox populations have substantially increased, especially in the densely populated and urbanized areas in many European regions, e.g., France (14), Switzerland (15), and Germany (16). There is strong evidence that these changes in the population dynamic of foxes led to a marked increase of the incidence of human AE in different regions of continental Europe during the last two decades (17, 18).

In parallel to this development, the parasite spread from historically known endemic regions in Central Europe like northern Switzerland, eastern France, southern Germany, and western Austria, over large distances toward the Baltic States (19, 20), Scandinavian countries (21, 22), and to the west of France (14, 23). In Switzerland, the southern border of the parasite distribution corresponds fairly well to the course of the Alpine crest (24). However, some case records of rodent infections south of the Alpine crest in Italy (25) and Switzerland (24, 26) demonstrate that the border of the distribution area is not just a result of the harsh climatic conditions in the high Alps. In the Swiss Canton Grison, a correlation between the prevalence in foxes and the predation of foxes on cricetid but not on murid species (26) gave evidence that, beside climatic factors (27), the distribution of suitable intermediate hosts is crucial for the distribution pattern of the parasite. In contrast to many other studies that suggest a geographical spread of *E. multilocularis*, recent investigations in Ticino, the most southern Canton of Switzerland which is located south of the Alpine crest, revealed a stable border of the distribution of the parasite over the last 20 years. Interestingly, its local distribution and its border of distribution matched the restricted areas where the vole *M. arvalis* was present (24).

Throughout Switzerland, nine different cricetid species occur, which could potentially act as intermediate hosts. However, only four of these species are wide spread and occur in higher densities (28). Two of the species, i.e., *A. scherman* and *M. arvalis*, live in open fields and have been described by different authors as important intermediate hosts in Central Europe (1). The prevalence of the widespread vole *M. agrestis*, a species living mainly in wetland, meadows, and young forests (29, 30), has less been studied. However, a recent investigation demonstrated its high susceptibility to experimental oral inoculations (31). Although no protoscoleces were found 6 weeks p.i. in this study, a recent field study in Sweden confirmed that this species can develop fertile infections (32). *Myodes glareolus*, the fourth widespread vole in Switzerland, lives mainly in bush lands and forests. It has been regularly described as potential intermediate host in Central Europe, but its epidemiological role for supporting or maintaining the parasite cycle remains unclear (1).

In order to effectively transmit the parasite in the European endemic area, the intermediate hosts have to develop fertile metacestodes with infective protoscoleces (33). In addition to being susceptible to the parasite, only species which share their habitat with foxes and are regularly predated by them can ensure the maintenance of the life cycle (34, 35). Therefore, prevalence studies do not suffice to compare the significance of different rodent species for the maintenance of the parasite life cycle. In addition, the direct comparison between different species is hampered by the fact that transmission intensity can greatly vary in space and time (35). Correspondingly, prevalence rates in rodent populations are strongly affected by changing environmental conditions over time and the infective state of the fox individuals in local fox territories.

With our study, we wanted to elucidate the relative importance of the vole species *A. scherman, M. arvalis, M. agrestis*, and *M. glareolus* in selected study plots where several of these vole species co-occur simultaneously. This should ensure (A) that the investigated rodents were most likely exposed to the feces of the same fox family groups (and therewith to the same overall level of environmental egg contamination) and (B) that the foxes of one family group could select between the different rodent prey species. To compare the relative importance of the investigated rodent species, we estimated for each species (1) the prevalence of *E. multilocularis*, (2) the proportion of fertile infections, (3) the asexual parasite reproduction (number of protoscoleces), and (4) the predation frequency by foxes.

## Materials and Methods

### Study Sites

**Figure 1:**
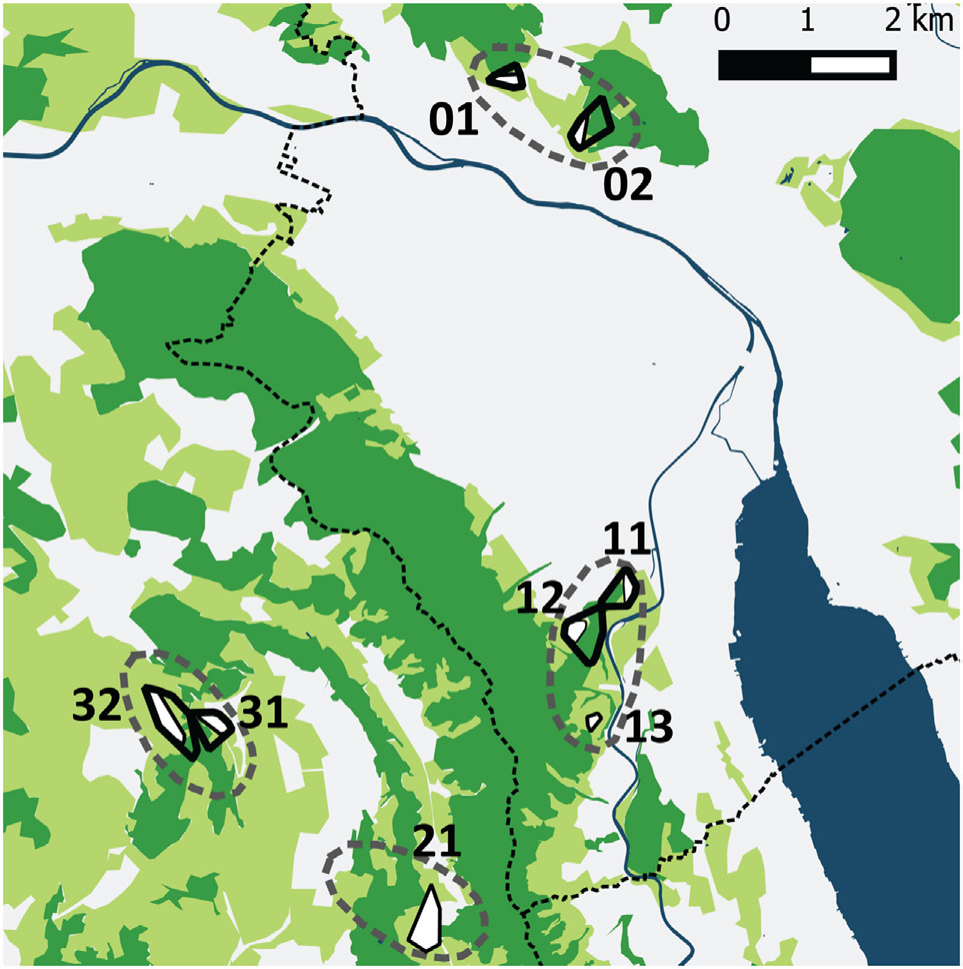
The four study sites (dashed lines) in the urban periphery of Zürich and the rural surroundings. Two sites were situated near to the city border and contained five trapping plots (01, 02 and 11, 12, 13) and two sites in rural settings contained three tapping plots (21 and 31, 32). The dotted line shows the border of the community of Zurich. The different colors represent water surfaces (dark blue), forests (dark green), cultivated (light green; mainly meadows and pastures), and urban areas (white). Black outlined polygons are the trapping plots for all four vole species, the white areas within these polygons are meadows and pastures where *Arvicola scherman* and *Microtus arvalis* were trapped. *Myodes glareolus* and *Microtus agrestis* have been trapped in the immediate neighborhood to these fields in scrubland and forest habitats.

The four study sites were located within and near the community of Zurich, which is situated in the Swiss midlands within a hilly landscape dominated by a mosaic of pastures, meadows, arable lands, and woodland and is characterized by a temperate climate (Köppen-Geiger climate classification Cfb, warm temperate, fully humid, warm summers) (36). Two study sites were situated along the periphery of the city of Zurich and two in rural settings in a distance of roughly 2–4 km from the border of the community (Figure 1). The altitude of the study sites is 400–600 m above sea level.

### Rodents Trapping and Analysis

*Arvicola scherman* and *M. arvalis* were trapped in meadows and pastures which is their preferred habitat. *M. agrestis* and *M. glareolus* which live in habitats with more cover, were trapped in field verges, scrublands, and forests in the direct neighborhood of the trapping fields for the former two species. Within each study site, one to three trapping plots were selected, which consisted of interconnected meadows, pastures, field verges, scrubland, and with adjoining forests. The borders of these plots were defined by a polygon that contained all trapped rodents. Thereby, *M. agrestis* and *M. glareolus*, which live in more covered habitats, were always attributed to the same plot as the nearest field with *A. scherman* and/or *M. arvalis*. The size of the eight study plots varied between 2 and 23 ha, and it was assumed that within a study site the same fox individuals had access to the different rodent species (home range sizes of resident foxes in Zurich according to Gloor (37): mean MCPs of 29 ha for females and 31 ha for males).

All rodents were trapped during five trapping seasons, namely during summer 2013 (August–September), fall 2013 (October), spring 2014 (mid-March–mid-June), fall 2014 (October–December), and spring 2015 (March–April). *A. scherman* and *M. arvalis* were trapped with un-baited Topcat traps (Topcat GmbH, Switzerland), which are well suited for the selective trapping of rodents that live in the open-field and move mainly in a system of runways and tunnels. The other two species, *M. agrestis* and *M. glareolus*, living in forest and scrublands, were trapped with live traps (Longworth, Penlon Ltd., Abingdon, UK) which were baited with cereals (bird food), apples, and straw. These traps were set for two consecutive nights and checked always early in the morning, at noon, and late in the evening. All unintentionally trapped small mammals were released. *M. agrestis* and *M. glareolus*, which were trapped alive, were euthanized by intraperitoneal injection of T61^®^ (Embutramid, Hoechst Veterinär, Unterschleißheim) after sedation. For each animal, coordinates, time, and date of capture were collected. All animals were stored at −20°C until further investigation.

Necropsy was carried out under a safety hood. Data on body length, weight (without abdominal organs), and sex were taken. The animals were categorized as reproducing or non-reproducing according to the development of the ductus deferens of the testes in males and placental scars or embryos in the uterus of females. Rodent species were determined according to Brohmer (38). *M. arvalis* and *M. agrestis* were distinguished by tooth examination (39). Livers were macroscopically examined for lesions. Suspicious lesions were isolated and investigated for protoscoleces. The number of protoscoleces was determined according to Stieger et al. (40). To compare the number of protoscoleces per individual between the investigated species, the records of fertile infections of the present study were complemented with corresponding data of two previous studies from Zurich (40, 41). All liver lesions without protoscoleces were collected for PCR analysis which was carried out according to Ref. (40) by detection of *E. multilocularis* DNA using a modified PCR (42) with a single primer pair [EM-H15 (5′-CCATATTACAACAATATTCCTATC-3′); EM-H17 (5′-GTGAGTGATTCTTGTTAGGGGAA-G-3′)].

### Feces Sampling and Analysis

During the trapping periods, all fox feces encountered in the trapping fields were collected and GPS coordinates were taken. After 5 days freezing at −80°C for safety reason, feces were stored at −20°C until further analysis. Hairs were collected while sieving 2 g of the feces for taeniid egg detection (43) and also from additional 2–5 g of the remaining fecal material. After washing and drying, hairs were analyzed for rodent prey determination by microscopic investigation after Teerink (44). For hair identification, we prepared prints of the cuticle and medulla on gelatin that had been spread as a thin layer on a microscope slide, and cut cross-sections using blades. Based on these investigations we could differentiate between the three vole genera, *Arvicola, Microtus*, and *Myodes*, and the murid genera *Apodemus*. However, the co-occurring species of the genera *Microtus* (i.e., *M. arvalis* and *M. agrestis*) and *Apodemus* (i.e., *A. sylvaticus* and *A. flavicollis*) could not be distinguished.

### Statistical Analysis

Prevalence rates of *E. multilocularis* and frequencies of infections with protoscoleces in rodents were analyzed using logistic regressions with the SPSS 22.0 (IBM) statistical software program. We chose six independent variables as possible factors for affecting prevalence rates and the proportion of fertile infections: SPECIES, REPRODUCTION, SEX, SEASON, PLOT, and WEIGHT. Weight was measured without abdominal organs and was used as a proxy of age. The comparison between the species was the main purpose of our investigations. Therefore, we used the data of a trapping plot during a specific season for the logistic regression only when a minimum of eight individuals of at least two species were available for this season.

Akaike’s information criterion (AIC, 76) was calculated based on the *K* value (degree of freedom + 2) and the −log likelihood, corrected for small sample size (AICc). The ΔAICc for all variations of the six variables was determined in order to define the best model with minimum influence between the variables. Models were only included in the selection procedure if all included variables significantly affected the model fit.

The non-parametric Kruskal–Wallis test for independent samples was used to compare the number of protoscoleces in different species. Predation rates on different rodent species were compared by calculating the exact binomial 95% confidence intervals (CI) for means of binomial variables, according to the method of Clopper and Pearson (45).

## Results

### Prevalence Rates

In this study, 1,918 voles were trapped and dissected, and liver lesions were detected in 430 individuals. In total, 49 of these individuals had fertile *E. multilocularis* infections with fully developed protoscoleces. The remaining 381 lesions were analyzed by PCR, whereof 91 samples were positive for *E. multilocularis*.

On a species level, the overall prevalence rates were 5.3% (95% CI 3.9–7.1, *N* = 810) in *A. scherman* and therewith less than half as in *M. arvalis* (11.0% CI 8.9–13.4, *N* = 773), whereas the prevalence of *M. glareolus* (3.9% CI 2.0–6.7, *N* = 306) was on a similar level as *A. scherman*. Although strong efforts have been taken to trap comparable numbers of *M. agrestis*, only 29 individuals were available for our dissections, and none of them was infected with *E. multilocularis* (0% CI 0–9.8, *N* = 29). We, therefore, focused in our further comparative analysis only on the other three species.

**Figure 2:**
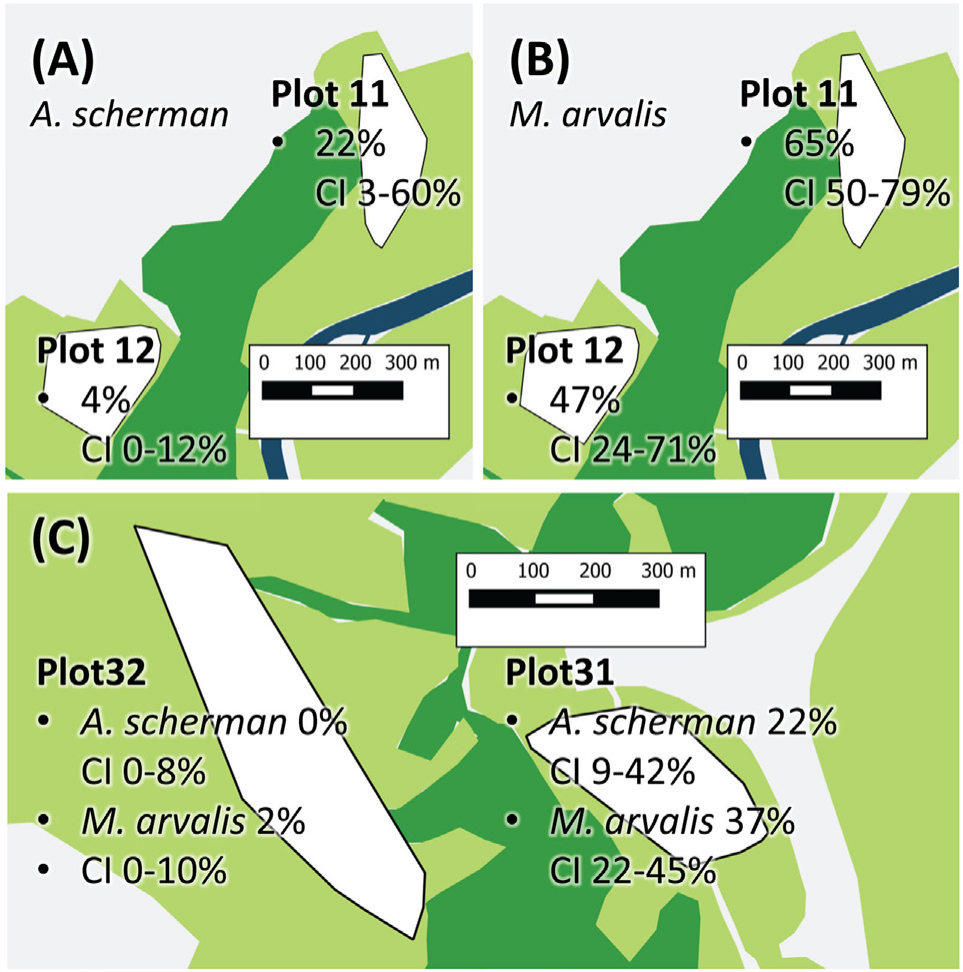
Exemplary prevalence rates of Plot 11 and 12 show *Arvicola scherman* **(A)** and *Microtus arvalis* **(B)** prevalences from spring 2014. On plot 12, the prevalence of *M. arvalis* is significantly higher compared to the prevalence of *A. scherman*. **(C)** The two vole populations of *A. scherman* and *M. arvalis*, are significant more frequently infected on plot 31 than on the nearby plot 32. The different colors represent forest (dark green), cultivated land (light green, mainly fields and pastures), and streets and village (light gray). The trapping plots for *A. scherman* and *M. arvalis* (white polygons) are situated in the cultivated land.

For building the multiple logistic regression models to explain prevalence rates and the frequency of fertile infections, data records of a given trapping plot and a given season were excluded if not at least data records of eight individuals per species for at least two species were available (see Materials and Methods). A total of 1,695 data records fulfilled this criterion. The best model to explain the prevalence of *E. multilocularis* contained all considered independent factors except the variable REPRODUCTION (Table 1). The model confirmed the results of the univariate comparison between the three species: The infection frequency for *M. arvalis* was significantly higher whereas the lower infection frequency of *M. glareolus* did not differ significantly from *A. scherman* (OR 2.69 and 0.57, respectively; Table 1). These differences could also be statistically validated within a single trapping field. Thus, during spring 2014, we recorded a prevalence of 47.4% (CI 24.4–71.1) for *M. arvalis* and 3.5% (CI 0.4–12.1) for *A. scherman* within the same pasture of trapping plot 12, and a similar trend was found in the neighboring plot 11 (Figures 2A,B).

**Table 1:** Odds ratios and the corresponding 95% CI of the best logistic regression models for (a) *Echinococcus multilocularis* prevalence in the most frequently trapped rodent species (*Arvicola scherman, Microtus agrestis* and *Myodes glareolus*) and (b) percentage of fertile infection (containing protoscoleces) out of all *Echinococcus multilocularis* positive rodents.

Independent factors^a^	*E. multilocularis* prevalence (*N* = 1695)		Protoscoleces prevalence (*N* = 132)	
	OR	95% CI	OR	95% CI
**SPECIES**				
*Microtus arvalis* vs. *Arvicola scherman*	2.69	1.66–4.37	11.23	3.49–36.08
*Myodes glareolus* vs. *A. scherman*	0.57	0.26–1.22	6.43	1.08–38.08

**SEASON**				
Summer13 vs. spring15	0.14	0.05–0.37	–	–
Fall13 vs. spring15	0.14	0.02–1.25	–	–
Spring14 vs. spring15	1.33	0.72–2.44	–	–
Fall14 vs. spring15	0.30	0.16–0.57	–	–

**PLOT**				
01 vs. 21	6.85	1.57–29.82	–	–
02 vs. 21	2.63	0.71–9.75	–	–
11 vs. 21	7.43	2.72–20.34	–	–
12 vs. 21	4.00	1.36–11.75	–	–
13 vs. 21	0.00^b^	–	–	–
31 vs. 21	4.48	1.61–12.44	–	–
32 vs. 21	1.03	0.32–3.36	–	–
SEX (female vs. male)	2.17	1.41–3.33	–	–
WEIGHT (without abdominal organs)	6.72	3.24–13.95	11.80	2.33–59.82
REPRODUCTION (yes/no)	–	–	–	–
Constant	0.00	–	0.01	–

*The factor REPRODUCTION did not enter the two best models*.

*^a^All possible combinations of the factors SPECIES, SEASON, PLOTS, SEX, WEIGHT, and REPRODUCTION were tested. In the model selection procedure based on the AICc, only models were considered in which all included factors showed a significant impact (*p* < 0.05)*.

*^b^No confidence interval (CI) could be calculated for plot 13 due to the small size of the sub-sample*.

In spring season, an increased percentage of infected rodents was observed which peaked up to 65% for *M. arvalis* on trapping plot 11 during spring 2014 (Figure 2B). Strong differences in prevalence rates were found between the trapping plots, with significant differences even between trapping fields in immediate neighborhood (Table 1). For example, the trapping fields for *M. arvalis* and *A. scherman* in plots 31 and 32 are separated by a small forest and lie in a distance of less than 300 m to each other (Figures 2,C). In spite of the short distance, we recorded much higher prevalences for *M. arvalis* and *A. scherman* in plot 31 during spring 2014 [*M. arvalis*: 37.8 (CI 22.5–55.2) vs. 2.0% (CI 0–10.4); *A. scherman* 21.4 (8.3–41.0) vs. 0.0% (0.0–15.3), respectively]. Interestingly, females were more frequently infected than males in all three species [*A. scherman*: male 3.7 (CI 1.9–6.4) vs. female 6.4% (CI 4.3–9.2); *M. arvalis*: 10.5 (CI 7.4–14.4) vs. 13.6% (CI 10.2–17.6), and *M. glareolus* 2.6 (CI 0.5–7.5) vs. 4.6% (CI 1.9–9.2)]. As expected, the logistic model confirms that individuals with higher weights were more likely to be infected with *E. multilocularis* [mean weights (without abdominal organs) of *A. scherman, M. arvalis*, and *M. glareolus* were 55.6 (SD ± 16.8), 16.7 (±5.3), and 16.9 (±3.5) g for non-infected and 70.0 (±12.0), 19.9 (±4.7), and 18.0 (±3.1) g for infected animals].

### Parasite Fertility and Protoscolex Burden

When analyzing the factors affecting whether an *E. multilocularis*-infected rodent had a non-fertile or fertile infection, only two of the six considered factors entered the best model. We detected a significantly higher probability for fertile infections in *M. arvalis* and *M. glareolus* than in *A. scherman* (OR 11.2 and 6.4, respectively; see Table 1). Non-fertile infected animals were more likely in lower weight classes than infected ones with fertile infections [mean weights (without abdominal organs including metacestode tissue) for *M. arvalis, A. scherman*, and *M. glareolus*: 69.5 (SD ± 11.6), 18.7 (±4.8), and 16.8 (±2.7) vs. 75.1 (±16.0), 21.2 (±4.3), and 20.4 (± 2.6) g, respectively].

**Figure 3:**
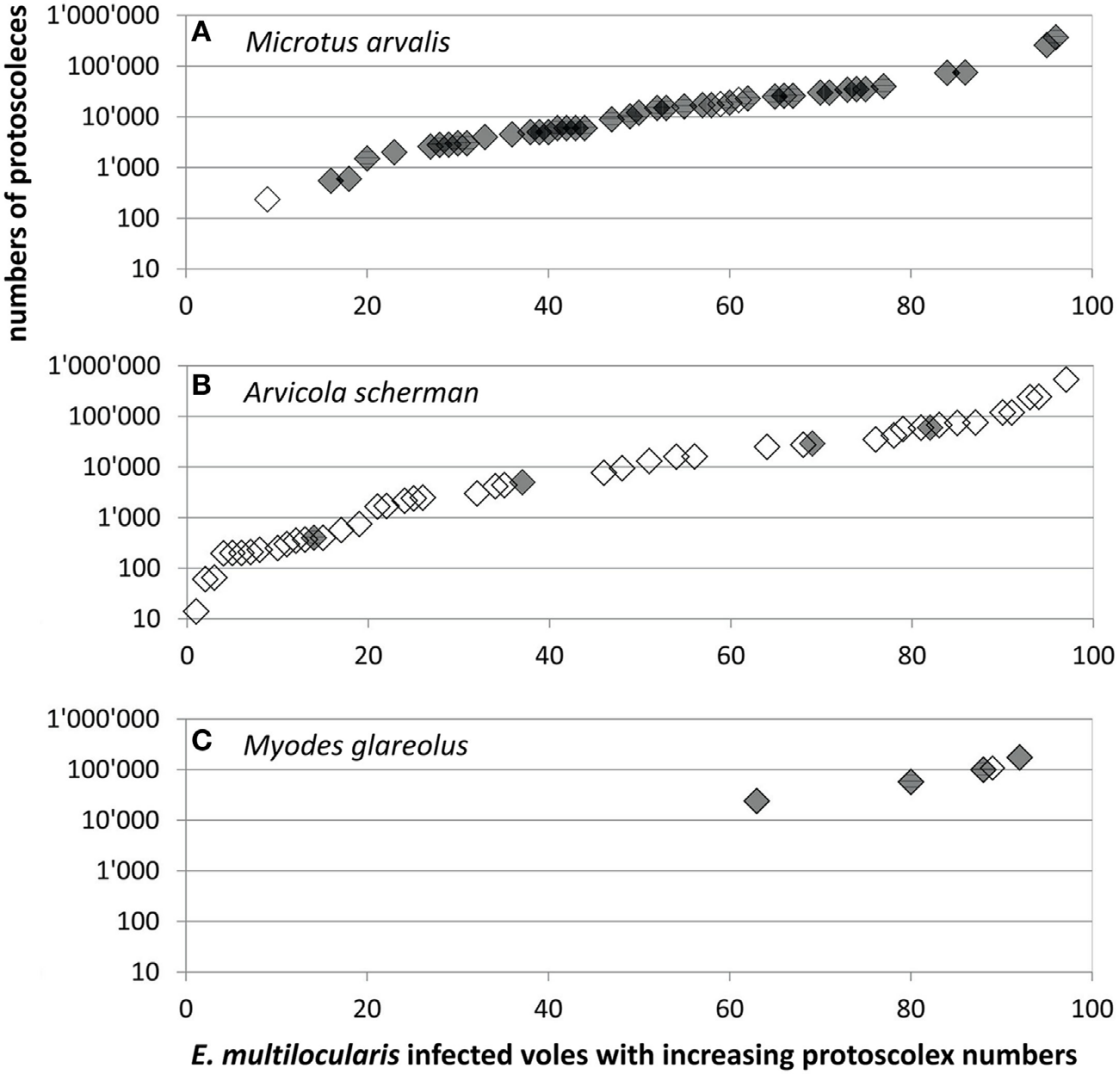
Protoscoleces burden in **(A)** 44 *Microtus arvalis*, **(B)** 46 *Arvicola scherman*, and **(C)** 5 *Myodes glareolus* with fertile *Echinococcus multilocularis* infections which were trapped for this (gray symbols, *N* = 49) and for former (blank symbols, *N* = 46) studies (40, 41). Data shown in Table S1 in Supplementary Material.

A total of 95 records were available for comparison of protoscoleces numbers according to the species, consisting of 49 records from this and another 46 from previous studies from Zurich and surrounding communities (see Materials and Methods). Not only the overall prevalence of *E. multilocularis* and the proportion of fertile infections but also the numbers of protoscoleces differed significantly between the vole species. The protoscolex burdens in five *M. glareolus* were 24,000, 57,600, 100,000, 108,000, and 175,000 (mean: 92,920, median: 100,000) and therewith significantly higher than in *M. arvalis* [range: 235–370,800, mean: 30,000, median: 13,500 (*N* = 44)] and *A. scherman* [range: 14–535,000, mean: 41,440, median: 4,290 (*N* = 46); adjusted H 9.3, df 2, *p* = 0.001; Figure 3].

### Predation of Foxes

In total 234 fecal samples of foxes, collected in the seven trapping plots where *E. multilocularis*-infected rodents have been found, were analyzed for the presence of different rodent prey species.

**Figure 4:**
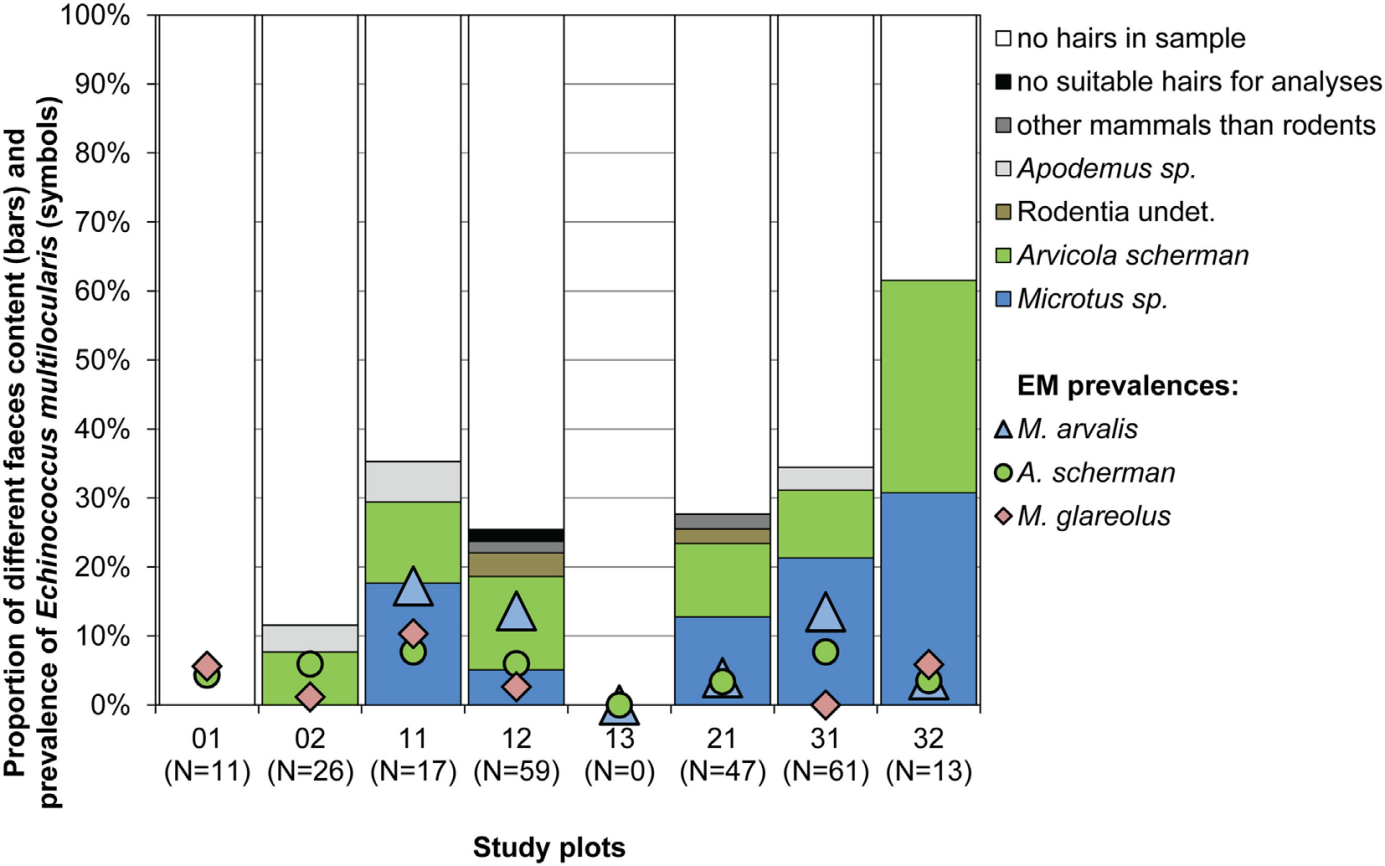
Proportion of different species determined by hair analyses of samples from 234 fox droppings (4–7 g per dropping) collected from spring 2014 until spring 2015 (bars) and percentages of *E. multilocularis*-infected voles per study plot and species during the whole study period (symbols). No *Myodes* sp. hairs were detected. No fecal analyses have been done for plot 13.

Hairs of prey species could be found in 66 samples, wherefrom 63 were identified as rodent hairs. As only 4–7 g of each feces was used to isolate hairs, only few hairs were available per sample. This can explain why we never detected more than one rodent genus within the same sample. The most abundant rodent prey remains were of the genera *Microtus* and *Arvicola* with 29 and 27 records, respectively [12.3 (CI 8.4–17.2) and 11.5% (CI 7.7–16.3)]. *Arvicola* was identified in six and *Microtus* in five of the seven investigated study plots (Figure 4). The genus *Apodemus* and non-identifiably rodent hairs were recorded only in four and three samples corresponding to 1.7 (CI 0.5–4.3) and 1.3% (CI 0.3–3.7). Interestingly, *M. glareolus* was never recorded as prey species (CI 0.0–1.3%), although the species has been trapped by us regularly in six study plots (Figure 4).

## Discussion

The emergence of AE across large regions of Europe has been associated with the increasing fox populations after the successful control of rabies in many European countries and an increased supply of anthropogenic food resources (1, 15, 18). However, the role and importance of different intermediate host species is under debate (1, 35, 46).

Investigations aiming to identify the key intermediate host species rely mostly on prevalence studies, and only a part of them also considers the parasite burden. In Europe, high prevalences have been found on a regular base in the four cricetid species *M. arvalis* [e.g., 18.6 (47), 3.0 (48), and 8.6% (49)], *A. scherman* [e.g., 13.6 (47), 3.6 (48), 6.5 (49), and 14.0% (50)], *M. glareolus* [e.g., 5.2 (5), 18.2 (48), 10.3 (49), and 4.4% (51)] and the muskrat *Ondatra zibethicus* [e.g., 22.1 (52), 1.6–62.5 (53), and 0.7% (54)]. Whereas the muskrat is rare in Zurich and, therefore, could not be analyzed in our study, our results confirm the relevance of the other three rodent species which all had relevant prevalence rates and a significant amount of fertile infections.

However, also other European cricetid species have been occasionally found infected like the sibling vole *Microtus levis* formerly *M. rossiaemeridionalis* in Svalbard [e.g., 18.9% (55)], *Chionomys nivalis* [syn. *Microtus nivalis*] in Romania (e.g., 77), *Microtus subterraneus* in France [1 infection among 169 individuals (56)], *Microtus agrestis* in France [1 infection among 16 individuals (5)] and in Sweden [1 infection among 187 individuals (32)], and *Arvicola amphibius* in Sweden [1.8% (32)]. Indeed, the example of Svalbard shows that *M. levis* can maintain the parasite life cycle and, therefore, this species possibly could play a significant role for the occurrence of the parasite in Eastern Europe. However, this species does not occur in the historically recognized endemic areas of France, Germany, Austria, Switzerland, and other countries of Western Europe. Considering that only in France one infected *M. subterraneus* (56) and one infected *M. agrestis* (5) and in Sweden few infected individuals of *M. agrestis* and *A. amphibius* (32) were found, it is very unlikely that these two species play a significant role in highly endemic regions. The same applies for *C. nivalis* as this species has a much more restricted distribution in Western Europe than *E. multilocularis*. However, it is known that *M. agrestis* is susceptible to experimental infections (31), and it replaces *M. arvalis* in the Scandinavian countries where the species expands more to open habitats than in regions where it co-occurs with *M. arvalis* (57). Therefore, it is likely that this species together with *A. amphibius* maintains the life cycle at least at a low level in Scandinavian countries where surprisingly so far no infected *M. glareolus* have been detected (58).

Although many studies have investigated different murid species for *E. multilocularis* infections (5, 40, 49, 51), to our knowledge there are so far only two confirmed cases of infected murid rodents in Western Europe, namely a *M. musculus* which was trapped in the cellar of an inhabited house in a small village in the French Auvergne (59) and a *R. norvegicus* with small, non-fertile lesions (5). Experimental studies have confirmed the very high resistance of laboratory rats (60) to inoculations with high numbers of *E. multilocularis* eggs, but elucidated that this resistance can be reduced with immunosuppressive interventions resulting in active infections (61). Therefore, this single case and few cases from Japan cannot be regarded as indicators for the intermediate host competence of *R. norvegicus*. Also other non-cricetid rodent species have been reported in Europe, as the introduced Nutria *Myocastor coypus* (53, 54) and the Eurasian beaver *Castor fiber* (62, 63), which both can harbor fertile infections (53, 62). Considering their potential to disperse over large distances and their longevity (64–66), these two species could occasionally be of some importance in the spread and the persistence of the parasite. However, both species live in low densities and are only occasionally reported in the fox diet [e.g., Ref. (67, 68)].

Based on a systematic review of epidemiological studies, Oksanen et al. (46) confirmed arvicolids (including the genera *Microtus, Arvicola*, and *Myodes*) and muskrats as important intermediate hosts for *E. multilocularis* in Europe. However, most of the studies included in this review did not consider to which extent the different rodent species were preyed on by final hosts. As muskrats are not a frequent prey of foxes, this species is regarded by other authors more as a bioindicator for the presence of the parasite rather than a key intermediate host (51, 54). Only in special cases, e.g., if trapped animals of control programs are left on river banks, the infective cadavers would be available in large numbers to foxes and boost the infection pressure (51).

By reviewing the existing literature, the three cricetid species *M. arvalis, A. scherman*, and *M. glareolus* can be regarded as the most important candidates for maintaining the parasite life cycle over large parts of its distribution area in Western Europe. All three species are widespread, can reach high population densities (28, 69), frequently co-occur in high endemic regions of Western Europe and have been regularly reported with fertile infections in the wild. In this study, we ensured by trapping different species in the same plots and during the same periods that the same fox families had access to the different rodent species. Thus, we can assume a similar overall exposition of the rodents to *E. multilocularis* eggs. Furthermore, foxes had the choice on which of these species the preferably prey. We also included *M. agrestis* in this comparative study, as this species is another common cricetid species in the Swiss midlands. However, despite of the huge trapping efforts we could catch only 29 individuals, and that none of these animals were infected. This gives evidence that this species can be neglected for maintaining the parasite life cycle in the high endemic region of the Swiss Midlands.

Comparing *M. arvalis, A. scherman*, and *M. glareolus* we found significant differences among the species on several levels. Interestingly, *M. arvalis* had—to our knowledge—the highest prevalence ever recorded which was considerably higher than the prevalence rates for *A. scherman* and *M. glareolus*. For example, during spring 2014, 28.6% of all trapped *M. arvalis* (95% CI: 22.4–35.4) and only 6.5% (3.6–10.7) of *A. scherman* and 2.6% (0.5–7.5) of *M. glareolus* were infected. The difference was even more pronounced when comparing only the fertile infections. In total, in 16.8% (95% CI: 11.9–22.8) of all trapped *M. arvalis* during the same time period were protoscoleces found, whereas the corresponding values were only 0.9% (0.1–3.3) for *A. scherman* and 0.9% (0.0–4.8) for *M. glareolus*. The eminent role of this species is also underlined by a recent experimental study which demonstrated the high susceptibility of *M. arvalis* for fertile *E. multilocularis* infections (70). Thereby, it has to be considered that *M. arvalis*, which is known for its short generation time, can reach very high population densities of more than 2,500 individuals per hectare (28), which is higher than the peak values for *A. scherman* and *Myodes* [*A. scherman*: >1,000 ind./ha; *M. glareolus*: 50–100 ind./ha (28, 69)]. Furthermore, *M. arvalis* are much smaller (20–35 g) than *A. scherman* [65–130 g (28)]. Therefore, a fox has to feed on several *M. arvalis* to have an equivalent of nutrition as from one *A. scherman*. It can also be assumed that *M. arvalis* is a much easier prey as it uses more superficial channels than *A. scherman*, which rarely leaves the tunnel system. This assumption is substantiated by the observation that *M. arvalis* is a preferred prey compared to other rodent species (71). Nevertheless, in our study both species have been detected in the fox feces in similar frequency.

Interestingly, our data indicate that *Myodes* is the best intermediate host in terms of fertility of the parasite. In experimental studies, its susceptibility to oral experimental infections was lower as compared with *Microtus* spp., but infected animals developed fertile infections (72). However, although *M. glareolus* is a widespread and common species, we recorded no predation on it by foxes in our study. This is in contrast to other studies in which foxes were shown to prey on *M. glareolus* (73, 74). The species lives—like *M. agrestis*—more in covered habitats and thus probably is less susceptible to fox predation (75). Taken together, we conclude that this species plays a minor role in the perpetuation of the life cycle in our study region. However, highly infected *Myodes* could occasionally be eaten by domestic dogs and thus contribute to the transmission of *E. multilocularis* to human.

Our study supports the evidence for a high relevance of *M. arvalis* [in accordance with Ref. (24); see Table 2]. However, it cannot be excluded that other European species can replace *M. arvalis*. Especially *A. scherman* has shown similar high prevalences in previous studies in Zurich. Thus, in one trapping plot an extraordinary high prevalence of 61% [95% CI (41–78)] was recorded for this species (41). It is possible that foxes prefer *Arvicola* when *M. arvalis* is not available or in very low densities. This is supported by a study of Weber and Aubry (78) in the Swiss Jura mountains where *A. scherman* was the main prey and recorded for 54.5% of the investigated prey items. Indeed, a sigmoid-like functional response to *A. scherman* density has already been described for the predation of foxes and the predation rate decreased when the density of *M. arvalis* increased (35, 79). On the other hand, *M. arvalis* was consumed at a high level even when its density was very low. However, to clarify to which extent such replacement processes buffer the life cycle would need further studies.

**Table 2:** Qualitative assessment on the relative importance of the investigated vole species for the transmission of *Echinococcus multilocularis* based on the results of this study.

Parameters	*Arvicola scherman*	*Microtus arvalis*	*Myodes glareolus*	*Microtus agrestis*
Prevalence rates^a^	++	+++	+	n.d.
Frequency of fertile infections^b^	+	+++	++	n.a.
Recorded parasite burdens^c^	+	+	++	n.a.
Predation rates^d^	++	++	−	n.d.

Overall relevance for transmission^e^	++	+++	+	−

*Symbols: −, not relevant; +, relevant; ++, highly relevant; +++, highest relevance; n.d., not detected; n.a., not applicable*.

*^a^*Microtus arvalis* had significantly higher prevalence rates than *A. scherman* and *M. glareolus* in this study. However, some former studies revealed also very high prevalence rates for *A. scherman* [e.g., Ref. (41)]. None of the 29 dissected *M. arvalis* was infected*.

*^b^We detected significantly higher probabilities for fertile infections in *M. arvalis* and *M. glareolus* than in *A. scherman*. Overall 41 of 773 *M. arvalis* (5.3%), 4 of 306 *M. glareolus* (1.3%), and 4 of 810 *A. scherman* (0.5%) had fertile infections*.

*^c^The protoscolex burdens in *M. arvalis* and *A. scherman* were on a similar level and significantly lower than in *M. glareolus* (see Figure 3)*.

*^d^The predation rate by foxes on *M. arvalis* and *A. scherman* were on a similar level whereas no predation has been detected on *M. glareolus* (see Figure 4)*.

*^e^Considering the high prevalence, the high parasite fertility and the frequent predation on M. arvalis, this species is supposed to have the highest relevance as intermediate host E. multilocularis transmission. Although prevalence and frequency of fertile infections are lower in A. scherman, the high predation rate on this species gives evidence for its importance for the life cycle. In contrast, the predation on M. glareolus seems to be very low which suggests that this species plays only a minor role as intermediate host. Presumably, the low trapping success on M. agrestis reflects a low abundance of this species. Furthermore, it lives like M. glareolus in covered habitats, which makes it more difficult to foxes to prey on this species than on M. arvalis and A. scherman which live in open habitats*.

In conclusion, our study highlights how differences between rodent species in their susceptibility, exposition to infective eggs, parasite fertility, and predation by foxes affect their relevance for the life cycle of *E. multilocularis*. Our results provide evidence that *M. arvalis* and probably to a lesser extent *A. scherman* distribution models could be good predictors for the distribution and abundance of *E. multilocularis* in Western Europe. Models on the distribution and abundance of these species, therefore, could allow to model parasite occurrence on a more detailed spatial scale than models on fox distribution, as foxes have a very broad and much more uniform distribution than the different rodent species.

## Ethics Statement

Trapping of animals was performed under the direct supervision of a veterinary specialist, and according to the Swiss law, the guidelines on Animal Welfare and the specific regulations of the Canton of Zurich (permit number 17/2013) by the Veterinary Office and the Ethics Committee of the Canton of Zurich (Kantonales Veterinäramt Zürich, Zollstrasse 20, 8090 Zürich, Switzerland).

## Author Contributions

All authors listed have made substantial, direct, and intellectual contributions to the work, and all approved its content for publication.

## Conflict of Interest Statement

The authors declare that the research was conducted in the absence of any commercial or financial relationships that could be construed as a potential conflict of interest.
