# Supplementary material for: Microtus arvalis and Arvicola scherman: Key Players in the Echinococcus multilocularis Life Cycle
Source: Front Vet Sci. 2017 Dec 13;4:216. doi: 10.3389/fvets.2017.00216 (PMC5733337; doi:10.3389/fvets.2017.00216)
Supplement: Supplementary file 1 [file table_1.PDF]

Supplementary Material

Microtus arvalis and *Arvicola scherman*: Key Players in the *Echinococcus multilocularis* Life Cycle

Olivia Beerli, Diogo Guerra, Laima Baltrunaite, Peter Deplazes, Daniel Hegglin*

*** Correspondence:** Daniel Hegglin: email@uni.edu

1 Supplementary Table

Supplementary Table 1. Numbers of protoscoleces in *Arvicola scherman*, *Microtus arvalis* and *Myodes glareolus* from this study and two previous studies (all conducted in Zurich and the near surroundings) which were used for the comparative analyses in the current study.

Species	Study	Year	Month of year	protoscolex burden
A. scherman	Stieger et al. 2002	2000	6	14
A. scherman	Burlet et al. 2011	2008	6	61
A. scherman	Stieger et al. 2002	1999	9	65
A. scherman	Burlet et al. 2011	2008	3	197
A. scherman	Stieger et al. 2002	2000	1	200
A. scherman	Stieger et al. 2002	2000	11	200
A.scherman	Burlet et al. 2011	2007	2	208
A.scherman	Burlet et al. 2011	2007	10	231
A.scherman	Stieger et al. 2002	2000	5	250
A.scherman	Stieger et al. 2002	2000	9	300
A.scherman	Stieger et al. 2002	2000	7	350
A.scherman	Burlet et al. 2011	2007	11	370
A.scherman	Stieger et al. 2002	2000	9	400
A.scherman	present study	2015	4	400
A.scherman	Burlet et al. 2011	2008	6	568
A.scherman	Burlet et al. 2011	2007	11	750
A.scherman	Stieger et al. 2002	2000	9	1'650
A.scherman	Stieger et al. 2002	2000	11	1'700
A.scherman	Stieger et al. 2002	1999	8	2'160
A.scherman	Stieger et al. 2002	1999	9	2'400
A.scherman	Burlet et al. 2011	2008	2	2'492
A.scherman	Stieger et al. 2002	2000	7	3'000
A.scherman	Burlet et al. 2011	2007	8	4'180
A.scherman	Stieger et al. 2002	2000	7	4'400
A.scherman	present study	2014	4	5'000

A.scherman	Stieger et al. 2002	2000	9	7'700
A.scherman	Stieger et al. 2002	2000	11	9'500
A.scherman	Stieger et al. 2002	2001	8	13'180
A.scherman	Stieger et al. 2002	1999	9	16'000
A.scherman	Stieger et al. 2002	2000	5	16'100
A.scherman	Burlet et al. 2011	2007	9	25'000
A.scherman	Stieger et al. 2002	2000	7	27'600
A.scherman	present study	2014	10	29'000
A.scherman	Stieger et al. 2002	2000	7	35'100
A.scherman	Stieger et al. 2002	2000	6	42'250
A.scherman	Stieger et al. 2002	2001	7	56'500
A.scherman	present study	2014	4	60'000
A.scherman	Burlet et al. 2011	2007	6	60'000
A.scherman	Burlet et al. 2011	2007	11	67'550
A.scherman	Stieger et al. 2002	2000	6	74'500
A.scherman	Stieger et al. 2002	2000	7	76'000
A.scherman	Stieger et al. 2002	2001	7	119'350
A.scherman	Stieger et al. 2002	1999	9	120'000
A.scherman	Stieger et al. 2002	2000	11	240'000
A.scherman	Stieger et al. 2002	2000	7	244'400
A.scherman	Stieger et al. 2002	2001	10	535'000
M. arvalis	Stieger et al. 2002	2001	2	235
M. arvalis	present study	2015	4	550
M. arvalis	present study	2014	3	600
M. arvalis	present study	2014	4	1'500
M. arvalis	present study	2015	4	2'000
M. arvalis	present study	2014	4	2'600
M. arvalis	present study	2014	4	2'800
M. arvalis	present study	2013	8	2'825
M. arvalis	present study	2014	3	3'000
M. arvalis	present study	2014	3	3'000
M. arvalis	present study	2014	3	4'000
M. arvalis	present study	2014	3	4'500
M. arvalis	present study	2014	3	5'000
M. arvalis	present study	2014	3	5'000
M. arvalis	present study	2014	3	5'000
M. arvalis	present study	2014	3	6'000
M. arvalis	present study	2014	3	6'000
M. arvalis	present study	2014	3	6'000
M. arvalis	present study	2014	10	6'000
M. arvalis	present study	2015	4	8'900
M. arvalis	present study	2014	3	10'200
M. arvalis	present study	2014	5	12'000

M. arvalis	present study	2014	4	15'000
M. arvalis	present study	2014	3	15'000
M. arvalis	present study	2014	3	16'000
M. arvalis	present study	2014	4	17'000
M. arvalis	present study	2014	3	17'000
M. arvalis	Stieger et al. 2002	2001	2	17'720
M. arvalis	present study	2014	3	19'000
M. arvalis	Stieger et al. 2002	2001	2	21'475
M. arvalis	present study	2014	3	23'000
M. arvalis	present study	2014	3	25'000
M. arvalis	present study	2014	4	26'000
M. arvalis	present study	2014	3	26'000
M. arvalis	present study	2014	3	30'000
M. arvalis	present study	2014	3	30'000
M. arvalis	present study	2015	4	34'300
M. arvalis	present study	2014	3	35'000
M. arvalis	present study	2014	3	35'000
M. arvalis	present study	2014	3	40'000
M. arvalis	present study	2014	3	74'000
M. arvalis	present study	2014	3	75'000
M. arvalis	present study	2014	11	260'000
M. arvalis	present study	2014	10	370'800
M. glareolus	present study	2014	11	24'000
M. glareolus	present study	2014	11	57'600
M. glareolus	present study	2014	6	100'000
M. glareolus	Stieger et al. 2002	2000	1	108'000
M. glareolus	present study	2014	11	175'000
